# *De novo* Synthesis of Sphingolipids Is Defective in Experimental Models of Huntington's Disease

**DOI:** 10.3389/fnins.2017.00698

**Published:** 2017-12-19

**Authors:** Alba Di Pardo, Abdul Basit, Andrea Armirotti, Enrico Amico, Salvatore Castaldo, Giuseppe Pepe, Federico Marracino, Fabio Buttari, Anna F. Digilio, Vittorio Maglione

**Affiliations:** ^1^IRCCS Neuromed, Pozzilli, Italy; ^2^Department of Drug Discovery and Development, Fondazione Istituto Italiano di Tecnologia, Genova, Italy; ^3^Institute of Biosciences and Bioresources, National Research Council, Naples, Italy

**Keywords:** HD, sphingolipid *de novo* biosynthesis, dhSph, dhS1P, dhCer, mass spectrometry, apoptosis

## Abstract

Alterations of lipid metabolism have been frequently associated with Huntington's disease (HD) over the past years. HD is the most common neurodegenerative disorder, with a complex pathogenic profile, typically characterized by progressive striatal and cortical degeneration and associated motor, cognitive and behavioral disturbances. Previous findings from our group support the idea that disturbed sphingolipid metabolism could represent an additional hallmark of the disease. Although such a defect represents a common biological denominator among multiple disease models ranging from cells to humans through mouse models, more efforts are needed to clearly define its clinical significance and the role it may play in the progression of the disease. In this study, we provided the first evidence of a defective *de novo* biosynthetic pathway of sphingolipids in multiple HD pre-clinical models. qPCR analysis revealed perturbed gene expression of sphingolipid-metabolizing enzymes in both early and late stage of the disease. In particular, reduction in the levels of sptlc1 and cerS1 mRNA in the brain tissues from manifest HD mice resulted in a significant decrease in the content of dihydroSphingosine, dihydroSphingosine-1-phospahte and dihydroCeramide [C18:0] as assessed by mass spectrometry. Moreover, *in vitro* studies highlighted the relevant role that aberrant sphingolipid metabolism may have in the HD cellular homeostasis. With this study, we consolidate the evidence of disturbed sphingolipid metabolism in HD and demonstrate for the first time that the *de novo* biosynthesis pathway is also significantly affected in the disease. This finding further supports the hypothesis that perturbed sphingolipid metabolism may represent a crucial factor accounting for the high susceptibility to disease in HD.

## Introduction

Huntington's disease (HD) is a rare monogenic hereditary condition characterized by degeneration of striatal and cortical tissues, associated with motor, cognitive and behavioral disturbances (Novak and Tabrizi, 2011). The disease is caused by an expansion of a poly-glutamine (poly-Q) tract in the N-terminal region of huntingtin (HTT), a ubiquitously expressed protein (Walker, 2007), whose mutant form may affect cellular homeostasis in and out the central nervous system (CNS) (Strong et al., 1993; Maglione et al., 2005, 2006a,b; Carroll et al., 2015).

Mutant HTT triggers a cascade of undesirable events ranging from early transcription dysregulation to metabolic alterations which are responsible for a complex clinical and molecular disease scenario (Clabough, 2013; Labbadia and Morimoto, 2013; Bates et al., 2015).

The recognition of deregulated lipid homeostasis is becoming increasingly evident in HD (Puri, 2001; Desplats et al., 2007; Valenza and Cattaneo, 2011; Handley et al., 2016). Over the last years, our group and others have extensively contributed to these findings by demonstrating that sphingolipid metabolism is aberrant in multiple preclinical models and in human post-mortem brain tissues from HD patients (Maglione et al., 2010; Di Pardo et al., 2016, 2017a; Pirhaji et al., 2016, 2017; Skene et al., 2017).

Sphingolipids represent the major lipid constituents of biological membranes and modulate several important functions for neuronal and non-neuronal cell populations (Gault et al., 2010; Sonnino et al., 2015). Sphingolipids govern essential physiological processes ranging from vascular and bone formation (Hla et al., 2008; Xiong and Hla, 2014; Holmes, 2015) to inflammatory response (Huang et al., 2013; Aoki et al., 2016), and regulate many of the molecular events crucial for brain development and neuronal survival (Mendelson et al., 2014; Van Echten-Deckert et al., 2014).

**Figure 1:**
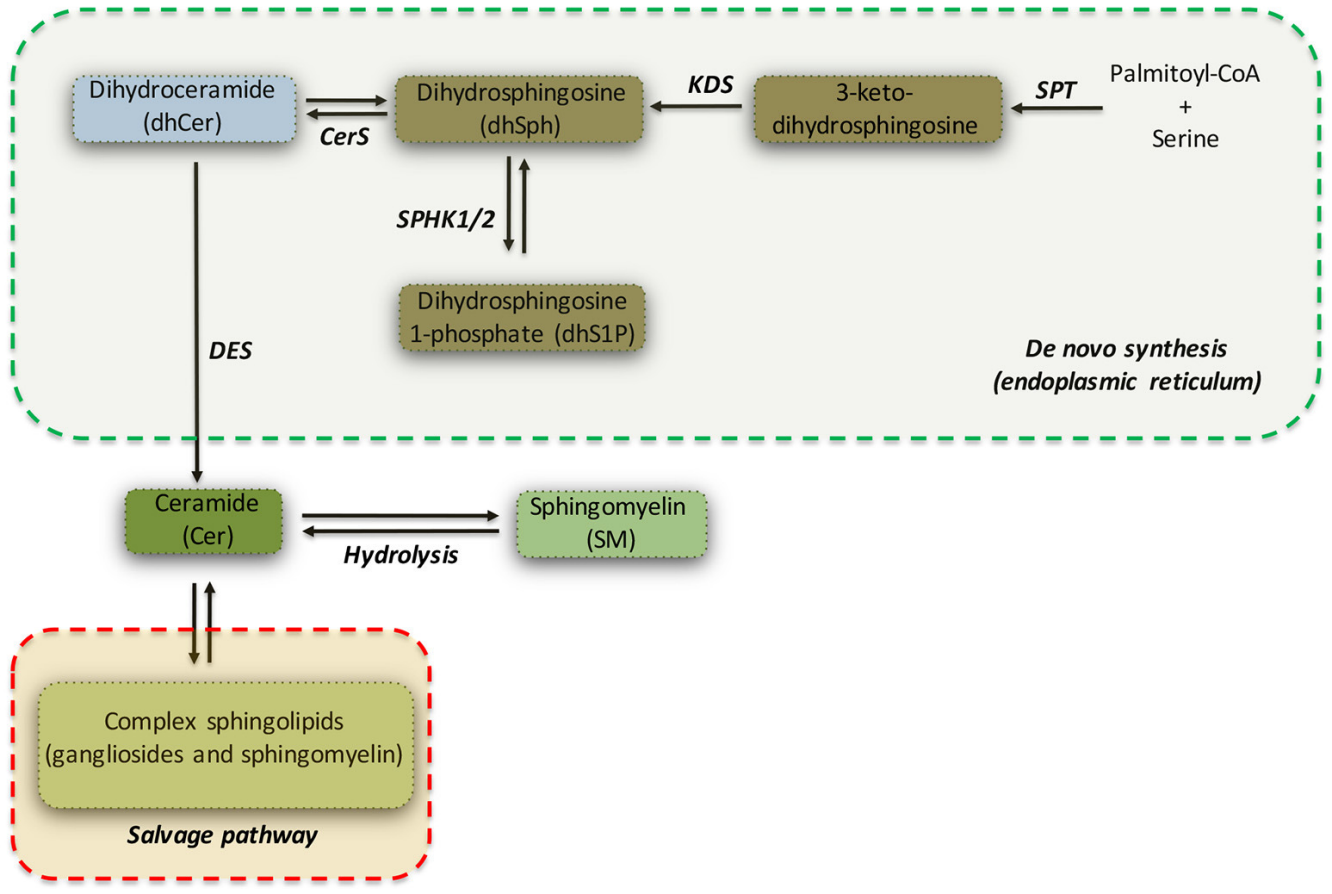
Simplified pathway of *de novo* sphingolipid synthesis. Serine palmitoyltransferase (SPT) catalyzes the initial reaction of the *de novo* biosynthesis of sphingolipids. Dihydrosphingosine (dhSPH) is generated after an intermediate step regulated by 3-keto-dihydrosphingosine reductase (KDS). Successively, dhSph can be either phosphorylated, with the generation of dhSphingosine-1-phosphate (dhS1P) by sphingosine kinase 1 and 2 (SPHK1/2), or acetylated by Ceramide Synthase (CerS) and desaturated by ceramide desaturase (DES) to form Ceramide (Cer). Ceramide may be also generated by both hydrolysis of sphingomyelin and by the *salvage pathway*.

Ceramide (Cer) can be considered as a metabolic hub in the sphingolipid biosynthesis and catabolism (see Figure 1) (Hannun and Obeid, 2008). It may be produced either by catabolism of complex sphingolipids (salvage pathway) or by *de novo* biosynthetic pathway (see Figure 1) (Gault et al., 2010), which takes place in the endoplasmic reticulum (ER).

The exact role of *de novo* synthesis of sphingolipids in the brain is not satisfyingly understood, however loss of expression or activity of the implicated enzymes is associated with a plethora of different deleterious events in both experimental models and human patients (Dawkins et al., 2001; Hojjati et al., 2005; Zhao et al., 2011; Ginkel et al., 2012; Vanni et al., 2014; Jun et al., 2015).

The initiating and rate-limiting step of *de novo* sphingolipid biosynthesis (Hanada, 2003) is catalyzed by serine palmitoyltransferase (SPT), a heterodimer composed of serine palmitoyltransferase long chain 1 and 2 (SPTLC1 and SPTLC2) subunits, both required for enzyme activity and with SPTLC2 as catalytic subunit (Hanada, 2003). SPT favors the condensation of serine with palmitoyl-CoA to form 3-keto-dihydrosphingosine (Hanada, 2003), which is subsequently reduced to form dihydrosphingosine (sphinganine or dhSph) by 3-keto-dihydrosphingosine reductase (KDS). dhSph may be then converted in either dihydrosphingosine-1-phosphate (dhS1P) by sphingosine kinases (SPHKs) or in dihydroceramide (dhCer) by ceramide synthases (CerS1-6). Finally, dihydroceramide desaturase (DES) catalyzes the final step of the *de novo* synthesis by converting dhCer in ceramide (see Figure 1).

Ceramide, thus generated, is transported to the Golgi complex, where it serves as a substrate for production of complex sphingolipids (Gault et al., 2010).

Levels of sphingolipids are particularly high in the nervous system (Giusto et al., 1992). Although sphingolipid recycling, by salvage pathway, seems to be predominant *in vitro* (Qin et al., 2010), a number of evidence highlights the critical role of *de novo* sphingolipid biosynthesis in neuronal function (Dawkins et al., 2001; Zhao et al., 2011; Vanni et al., 2014).

In this study, we provided the first evidence of defective *de novo* synthesis of sphingolipids in multiple HD experimental models. Our findings demonstrated that aberrant gene expression of “sphingo-biosynthetic” enzymes was associated with lower bioavailability of different sphingolipids in brain tissues from transgenic HD mouse model (R6/2). Furthermore, our *in vitro* results also suggested that such defective *de novo* synthesis may represent an important determinant in the pathogenesis of the disease and significantly contribute to its highly complex pathogenic profile.

## Materials and methods

### Mouse model

Mouse colonies were maintained in the animal facility at IRCCS Neuromed. All animal studies were performed in accordance with approved protocols by the IRCCS Neuromed Animal Care Review Board and by “Istituto Superiore di Sanità” (permit number: 1163/2015-PR) and were conducted according to EU Directive 2010/63/EU for animal experiments. Analyses were performed on early manifest (6 week old mice) and manifest (11 week old) R6/2 HD mice **(F1-F3)** and age-matched wild-type (WT) littermates.

### Quantitative real time PCR (qRT-PCR)

Mice were sacrificed by cervical dislocation and brain regions were dissected out, snap frozen in liquid N2 and pulverized in a mortar with a pestle. Total RNA was extracted using RNeasy kit (Qiagen) according to the manufacturer's instructions. One microgram of total RNA was reverse-transcribed using Superscript II reverse transcriptase (Invitrogen) and oligo-dT primer, and the resulting cDNAs were amplified using Power SYBR Green PCR Master Mix (Applied Biosystems) following the manufacturers' instructions. Quantitative PCR analysis was performed on a StepOne instrument (Applied Biosystems) as previously described (Di Pardo et al., 2017b). The following primers were used (5′–>3′): sptlc1 Fw-TACTCAGAGACCTCCAGCTG; sptlc1Rv: CACCAGGGATATGCTGTCATC; sptlc2Fw-GGAGATGCTGAAGCGGAAC; sptlc2Rv-GTATGAGCTGCTGACAGGCA; cers1Fw-CACACATCTTTCGGCCCCT; cers1Rv-GCGGGTCATGGAAGAAAGGA. Expression of sphingolipid-metabolizing enzymes was normalized on Cyclophilin-A by using the following primers: CycAFwd -TCCAAAGACAGCAGAAAACTT TCG; CycARv-TCTTCTTGCTGGTCTTGCCATTCC.

### Mass spectrometry (MS)

Snap frozen brain tissues were transferred into glass tubes and stored at −80°C until lipid extraction. Lipids were extracted using a modified Bligh and Dyer method as previously described (Basit et al., 2015; Di Pardo et al., 2017a).

### Cell models

Conditionally immortalized mouse striatal knock-in cells expressing endogenous levels of wild-type (WT) (STHdh^7/7^) or mutant Htt (STHdh^111/111^) were purchased from the Coriell Cell Repositories (Coriell Institute for Medical Research, Camden, NJ, USA) and maintained as previously described (Maglione et al., 2010).

### Treatment with myriocin and analysis of apoptosis

Cells were cultured in standard growth medium for 36 h in the presence and absence of 50 μM myriocin (Santa Cruz, Cat. N. sc-201397) and then maintained under apoptotic conditions (5 h in serum free medium at 39°C) as previously described (Di Pardo et al., 2014). For the analysis of apoptosis, at the end of the treatment, cells were collected and incubated with FITC-conjugated Annexin V (BD, Cat. N. 556419), according to the manufacturer's instructions. Fluorescence-activated cell sorting (FACS) analysis was performed as previously described (Di Pardo et al., 2014).

### Statistics

All data were expressed as mean ± SD. Statistical significance was calculated by Two-tailed Unpaired *t*-test using Prism 4.0 software (GraphPad Software). *p* < 0.05 were considered significant.

## Results

### Expression of enzymes involved in the *de novo* synthesis of sphingolipids is defective in multiple HD experimental models

**Figure 2:**
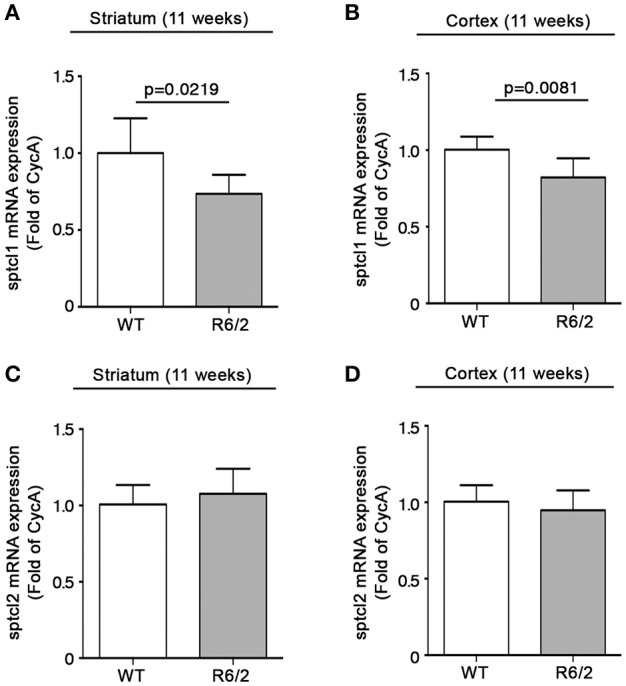
Expression of SPT subunit sptcl1 is reduced in brain tissues from R6/2 mice. qPCR analysis of sptcl1 and 2 in striatal **(A,C)** and cortical **(B,D)** tissues from manifest (11-week old) R6/2 mice and wild type littermates. Values are represented as mean ± SD. *N* = 6–8 for each group of mice. For statistical analyses, Two-tailed Unpaired *t*-test was used; *p* < 0.05 were considered significant.

**Figure 3:**
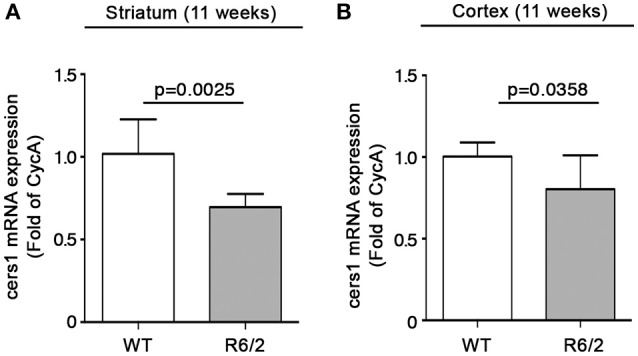
Expression of cers1 is reduced in brain tissues from R6/2 mice. qPCR analysis of cers1 in striatal **(A)** and cortical **(B)** tissues from manifest (11-week old) R6/2 mice and wild type littermates. Values are represented as mean ± SD. *N* = 6–8 for each group of mice. For statistical analyses, Two-tailed Unpaired *t*-test was used; *p* < 0.05 were considered significant.

We have recently reported that sphingolipid metabolism is aberrant in HD (Di Pardo et al., 2017a). Here, in order to provide a clearer picture of the deranged sphingolipid homeostasis in the disease, we investigated any potential alteration of the *de novo* biosynthetic pathway of these lipids. In particular, we assessed the expression profiles of sptlc1 and sptlc2 as well as of cers1 gene which normally synthesizes C18 chain-length-specific dhCer [dhCer (C18:0)], in both striatum and cortex of manifest HD mice. qPCR analysis showed a significant reduction of mRNA expression levels of sptlc1 in HD mice with respect to WT littermates in both striatum (1.021 ± 0.2262 vs. 0.7553 ± 0.1246; *p* = 0.0219, Unpaired *t*-test; Figure 2A) and cortex (1.016 ± 0.1131 vs. 0.8225 ± 0.12149; *p* = 0.0081, Unpaired *t*-test; Figure 2B). Similar results were obtained for cers1 in either striatal (0.6957 ± 0.08041 vs. 1.018 ± 0.2090; *p* = 0.0025, Unpaired *t*-test; Figure 3A) or cortical regions (1.003 ± 0.08614 vs. 0.8026 ± 0.2075; *p* = 0.0358, Unpaired *t*-test; Figure 3B). No changes in any of the two brain regions were observed for sptlc2 mRNA levels (Figures 2C,D). Moreover, defective gene expression of both SPT subunits was also observed in two fly models of the disease (Supplementary Figure 1).

### Content of *de novo* synthesized sphingolipids is reduced in brain tissues of manifest R6/2 mice

**Figure 4:**
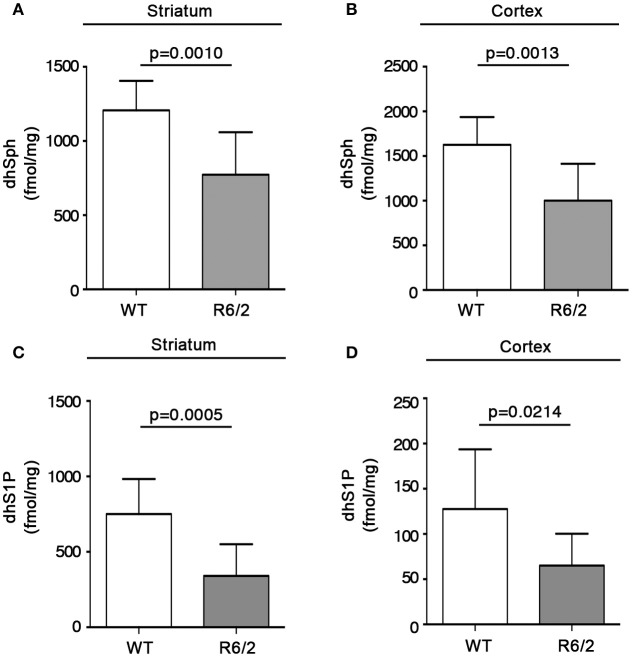
Levels of dhSph and dhS1P are reduced in brain tissues from R6/2 mice. Lipidomic analysis by LC-MS/MS of dhSph and dhS1P in striatal **(A,C)** and cortical **(B,D)** tissues from manifest (11-week old) R6/2 mice and wild type littermates. Values are represented as mean ± SD. *N* = 7–10 for each group of mice. For statistical analyses, Two-tailed Unpaired *t*-test was used; *p* < 0.05 were considered significant.

**Figure 5:**
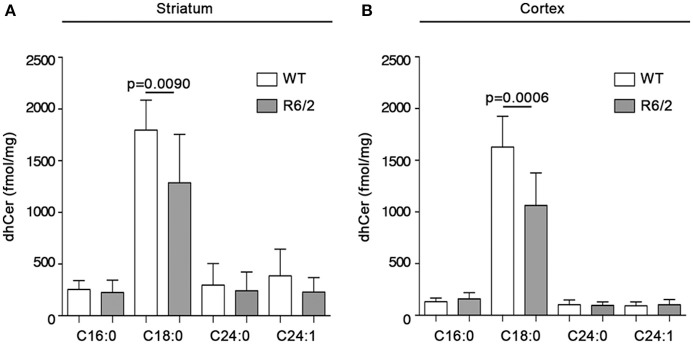
Levels of dhCer (C:18) are reduced in brain tissues from R6/2 mice. Lipidomic analysis by LC-MS/MS for different species of dhCer (C16:0, C18:0, C24:0, C24:1) in striatal **(A)** and cortical **(B)** tissues from manifest (11-week old) R6/2 mice and wild type littermates. Values are represented as mean ± SD. *N* = 7–10 for each group of mice. For statistical analyses, Two-tailed Unpaired *t*-test was used; *p* < 0.05 were considered significant.

With the aim of exploring the possibility that reduced gene expression of the metabolizing enzymes, SPT and cers1, could affect the bioavailability of sphingolipids deriving from *de novo* synthesis in mouse brains, quantitative analysis by MS was performed in both striatum and cortex from manifest R6/2 mice. Interestingly, altered gene expression was associated with a significant reduction in the content, expressed as fmol/mg tissue, of *de novo*-synthesized sphingolipids in R6/2 mice with respect to WT littermates. In particular, dhSph content was significantly reduced in both striatum (1207 ± 198.5 vs. 773.1 ± 286.7; *p* = 0.0010, Unpaired *t*-test; Figure 4A) and cortex (1625 ± 310.3 vs. 1001 ± 413.7; *p* = 0.0013, Unpaired *t*-test; Figure 4B). Similar decrement was observed for dhS1P levels in both striatum (750.6 ± 232.8 vs. 340.9 ± 209.7; *p* = 0.0005, Unpaired *t*-test; Figure 4C) and cortex (127.7 ± 65.99 vs. 65.08 ± 35.31; *p* = 0.0214, Unpaired *t*-test; Figure 4D). Analysis of different ceramide species revealed the reduction of only dhCer (C18:0) in both brain regions (striatum: 1797 ± 290.4 vs. 1286 ± 468.7; *p* = 0.0090, Unpaired *t*-test; Figure 5A) (cortex: 1628 ± 297.8 vs. 1064 ± 315.0; *p* = 0.0006, Unpaired *t*-test; Figure 5B) from the same manifest HD mice.

### *De novo* sphingolipid metabolism is aberrant at early stage of the disease in brain tissues from R6/2 mice

In order to assess any possible alteration related to the *de novo* biosynthesis of sphingolipids occurring early in the disease, and with the aim to understand whether it may eventually represent a precocious biological event in the pathogenesis of the disease, we investigated mRNA expression profiles of sptlc1, sptlc2 and cers1 in both striatal and cortical tissues from early manifest (6 week old) R6/2 mice and age-matched WT littermates.

**Figure 6:**
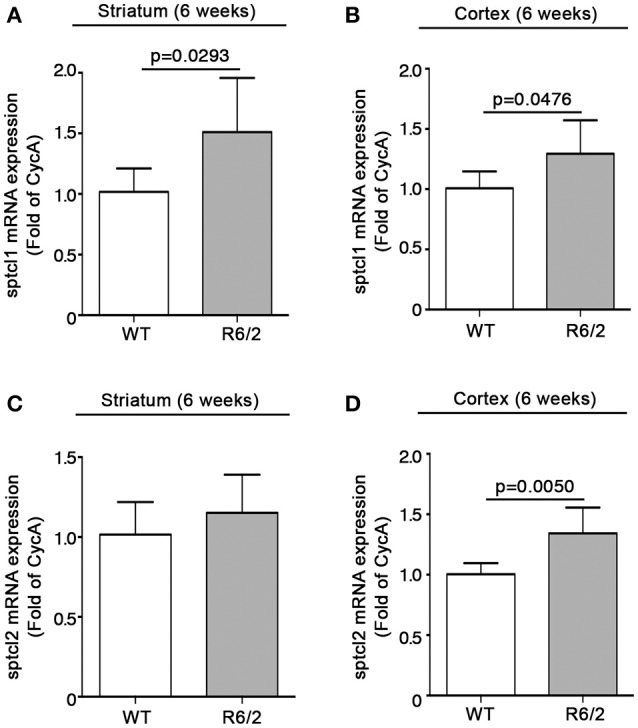
Expression of SPT subunits sptlc1 and sptlc2 is increased in brain tissues from early manifest R6/2 mice. qPCR analysis of sptcl1 and 2 in striatal **(A,C)** and cortical **(B,D)** tissues from early manifest (6-week old) R6/2 mice and wild type littermates. Values are represented as mean ± SD. *N* = 6 for each group of mice. For statistical analyses, Two-tailed Unpaired *t*-test was used; *p* < 0.05 were considered significant.

Sptlc1 mRNA levels were significantly increased in HD mice with respect to WT littermates in both brain regions (striatum: 1.018 ± 0.1931 vs. 1.511 ± 0.4457; *p* = 0.0293; Unpaired *t*-test; Figure 6A) (cortex: 1.008 ± 0.1397 vs. 1.295 ± 0.2780, *p* = 0.0476, Unpaired *t*-test; Figure 6B). Expression of sptlc2 was, significantly increased only in the cortex of the same animals (1.004 ± 0.09274 vs. 1.343 ± 0.2126; *p* = 0.0050, Unpaired *t*-test; Figures 6C,D).

**Figure 7:**
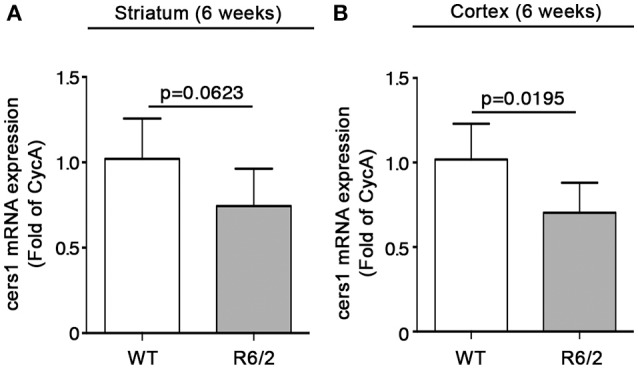
Expression of cers1 is reduced in brain tissues from early manifest R6/2 mice. qPCR analysis of cers1 in striatal **(A)** and cortical **(B)** tissues from early manifest (6-week old) R6/2 mice and wild type littermates. Values are represented as mean ± SD. *N* = 6 for each group of mice. For statistical analyses, Two-tailed Unpaired *t*-test was used; *p* < 0.05 were considered significant.

Conversely, mRNA levels of cers1 were slightly but not significantly reduced in the striatum (1.020 ± 0.2365 vs. 0.7442 ± 0.2192; *p* = 0.0623; Unpaired *t*-test; Figure 7A) and robustly decremented in the cortex (1.018 ± 0.2124 vs. 0.7032 ± 0.1779; *p* = 0.0195, Unpaired *t*-test; Figure 7B).

### Pharmacological inhibition of SPT activity increases cell susceptibility to apoptosis in ST*Hdh*^7/7^ to the same extent as ST*Hdh*^111/111^ cells and affects expression of sptlc1, sptlc2, and cers1 genes

In order to test the hypothesis that altered *de novo* sphingolipid synthesis might play a pivotal role in the control of cellular homeostasis and survival in HD cells, WT (ST*Hdh*^7/7^) cells, which normally display lower susceptibility to apoptosis (Maglione et al., 2010; Di Pardo et al., 2014) than HD (STHdh^111/111^) cells, were treated with myriocin, a selective SPT inhibitor (Hanada et al., 2000).

**Figure 8:**
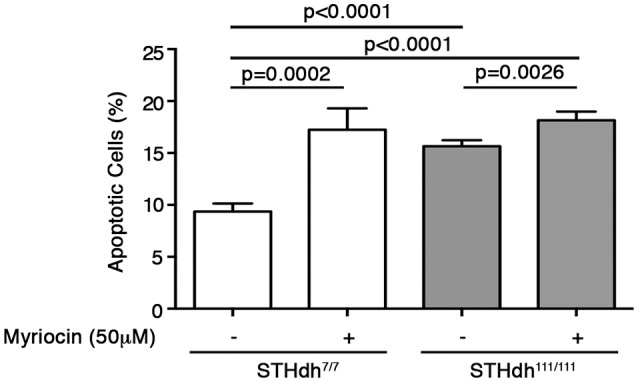
Pharmacological inhibition of SPT increases cell susceptibility to apoptosis in wild type STHdh^7/7^ cells to the same extent as HD STHdh^111/111^ cells. Analysis of apoptosis in STHdh cell lines first cultured for 36 h in complete medium in supplemented or not with 50 μM myriocin and then incubated for 5 h in serum-free medium to induce apoptosis. Values are mean ± SD of two experiments performed in triplicate. For statistical analyses, Two-tailed Unpaired *t*-test was used; *p* < 0.05 were considered significant.

**Figure 9:**
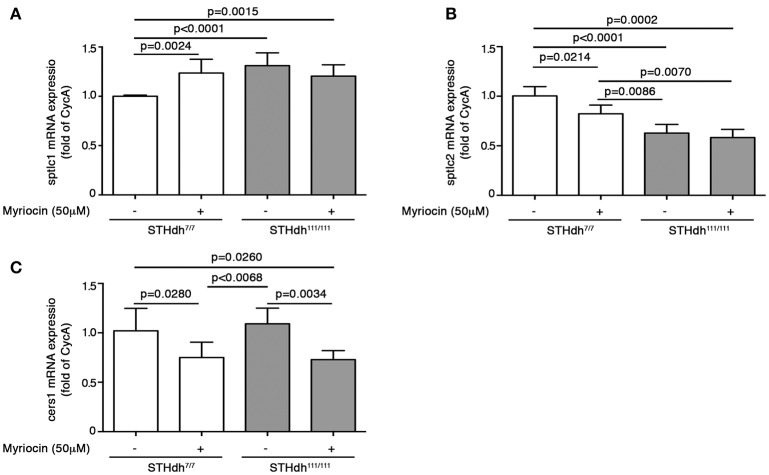
Pharmacological inhibition of SPT modulates gene expression of *de novo* biosynthetic enzymes in STHdh cells. qPCR analysis of sptlc1 **(A)**, sptlc2 **(B)**, and cers1 **(C)** in STHdh cell lines cultured for 36 h in complete medium supplemented or not with 50 μM myriocin. Values are mean ± SD of two independent experiments performed in duplicate or triplicate. For statistical analyses, Two-tailed Unpaired *t*-test was used; *p* < 0.05 were considered significant.

To this purpose, cells were cultured for 36 h in complete growth medium supplemented or not with 50 μM myriocin and then maintained under apoptotic conditions as previously described (Di Pardo et al., 2014). Annexin V-FITC staining was then performed to assess percentage of cell death. Interestingly, treatment with myriocin increased WT ST*Hdh*^7/7^ cell susceptibility to apoptosis to the same extent as HD STHdh^111/111^ cells (17.25 ± 2.047 vs. 15.66 ± 0.5784; *p* = 0.1809, Unpaired *t*-test; Figure 8) and markedly influenced gene expression of all three enzymes implicated in the *de novo* sphingolipid metabolism. In particular, the myriocin-mediated higher vulnerability to apoptosis in WT cells was associated with increased mRNA levels of sptlc1 to the same extent as HD cells (1.236 ± 0.1394 vs. 1.311 ± 0.1294; *p* = 0.3458, Unpaired *t*-test; Figure 9A) and reduced mRNA levels of sptlc2 (1.000 ± 0.01215 vs. 0.8235 ± 0.08730; *p* = 0.0214, Unpaired *t*-test; Figure 9B) and cers1 (1.020 ± 0.2276 vs. 0.7510 ± 0.1547; *p* = 0.0280; Unpaired *t*-test; Figure 9C). Conversely, in HD cells, myriocin only induced a merely detectable increase in the percentage of apoptotic cells with only gene expression of cers1 responsive to the treatment (Figure 9).

## Discussion

Evidence indicates that defective sphingolipid metabolism may likely contribute to different neurodegenerative conditions, including HD (Desplats et al., 2007; Maglione et al., 2010; Ceccom et al., 2014a,b; Couttas et al., 2014, 2016; Di Pardo et al., 2016, 2017a). We and others have recently demonstrated that alterations in the sphingolipid metabolism occur early in the disease stage and may play, therefore, a critical role in the pathogenesis of HD (Di Pardo et al., 2016, 2017a; Skene et al., 2017).

In this study, we provide the first evidence of altered *de novo* biosynthesis of sphingolipids in HD. In particular, we described for the first time an aberrant gene expression of all the analyzed biosynthetic enzymes-cers1, sptlc1 and sptlc2-in different experimental models of the disease.

Cers1 is particularly affected in this context. Reduction of mRNA levels was already detectable early in HD and persisted throughout the course of the disease.

To date, the significance of this alteration is not yet completely clear, however current available literature suggests a critical role in different pathological conditions in both animal models and humans. Lost activity of CerS1 has been reported to trigger early-onset cerebellar ataxia and cerebellar Purkinje cell degeneration in mice (Zhao et al., 2011; Ginkel et al., 2012). Absence of CerS1 in knockout mice is, indeed, associated with reduced levels of gangliosides (Ginkel et al., 2012; Spassieva et al., 2016) and myelin associated glycoprotein (MAG) (Ginkel et al., 2012), two molecular features that have been reported also to characterize HD pathology (Desplats et al., 2007; Maglione et al., 2010; Di Pardo et al., 2012, 2014, 2016). In humans, reduced CerS1 activity has been associated with myoclonus epilepsy and dementia (Vanni et al., 2014), while mutation in SPT gene, the rate-limiting enzyme of the *de novo* synthesis, causes a hereditary peripheral neuropathy (Dawkins et al., 2001).

Here, we demonstrate that gene expression of sptlc1 subunit is also reduced in brain tissues from HD mice at late stage of the disease. Such a defect may conceivably affect SPT enzymatic activity, and along with altered gene expression of cers1, may be likely responsible for the abnormal levels of sphingolipids in manifest R6/2 mice. Indeed, quantitative analysis by mass spectrometry revealed a significant reduction in the content of different sphingolipids in the brain tissues from the same mice. Reduction in the levels of dihydrosphingosine, the first sphingolipid synthesized in the *de novo* biosynthetic pathway, was associated with a marked decrement of dhS1P content and followed by a significant reduction in dhCer (C18:0) levels.

The biological role of dhS1P remains to be defined, however it is well-known that it binds S1P receptors similarly to S1P (Im et al., 2001). Elevation of its levels results beneficial in different disease models (Bu et al., 2010; Gorshkova et al., 2013; Nguyen-Tran et al., 2014) and is likely to contribute to the therapeutic action of FTY720 (fingolimod) (Berdyshev et al., 2009), a sphingomimetic drug FDA-approved for the treatment of Multiple Sclerosis (La Mantia et al., 2016), with neuroprotective properties also in different models of brain diseases including HD (Deogracias et al., 2012; Hemmati et al., 2013; Takasugi et al., 2013; Di Pardo et al., 2014; Fukumoto et al., 2014; Miguez et al., 2015; Potenza et al., 2016; Vidal-Martinez et al., 2016). dhS1P, similarly to S1P, has been also described to act as an endogenous inhibitor of histone deacetylases (HDACs) (Hait et al., 2009, 2014), whose activities have been widely described to be significantly incremented in HD models and their selective inhibition is currently identified as a promising therapeutic approach for the treatment of the disease (Sadri-Vakili and Cha, 2006).

Studies revealed that dhCer may trigger both physiological and pathological cellular responses (Siddique et al., 2015). Although the majority of these studies refers to undesirable effects of accumulation of dhCer, it cannot be excluded the possibility that even its reduction may have similar effects.

Whether reduction in these sphingolipids is a consequence of decreased bioavailability of dihydrosphingosine or depends on defective activity and/or expression of specific metabolizing enzymes (see Figure 1) is still unknown and more investigation is needed.

An important finding of our study is the evidence that defects in the expression of *de novo* sphingolipid biosynthetic enzymes occur early in the disease in HD mice. While mRNA expression of cers1 was markedly reduced, sptlc1 and 2 genes displayed an opposite expression profile. Although only speculative, since no quantitative data of the levels of *de novo* synthesized sphingolipids at this stage of the disease is available at moment, we believe that increased levels of mRNA for the two subunits may represent a compensatory strategy to counteract the hypothetical precocious reduction of *de novo* sphingolipid content. This hypothesis is well supported by our previous evidence that content of certain salvage pathway-derived sphingolipid species (see Figure 1) is already abnormal at early stages of the disease in brain tissues from R6/2 mice (Di Pardo et al., 2016, 2017a). From the mechanistic point of view, although not investigated in this study, it is plausible that at this stage of the disease, HTT mutation may have limited negative effect and interfere with normal SPT activity, coherently with the evidence indicating that SPT inhibition may determine an increase of sptlc1 mRNA levels (see Figure 9) (Bernhart et al., 2015). On the other hand, as the disease progresses, mutant huntingtin may have a generalized toxic effect and therefore determine a significant reduction of gene expression of the enzymes as found in manifest R6/2 mice.

The novelty of this study lies also on the evidence that alteration in the activity of these metabolizing enzymes and the subsequent perturbation of *de novo* sphingolpid synthesis, may represent key molecular mechanisms underlying the increased cell-death susceptibility in HD (Maglione et al., 2010; Di Pardo et al., 2014, 2017a). Interestingly, myriocin-mediated inhibition of SPT activity in WT cells (STHdh^7/7^) resulted in an increased vulnerability of these cells to the extent they become undistinguishable from HD cells. Although only speculative, the toxic effects of SPT inhibition in STHdh^7/7^ cells may be likely attributable to the plausible reduction of synthesized sphingolipids.

All the enzymes involved in the *de novo* synthesis of sphingolipids normally reside in the ER (Breslow, 2013; Siow et al., 2015), which has long been strongly implicated in the pathology of HD (Jiang et al., 2016; Jung et al., 2017). Whether the dysfunctional sphingolipid synthesis reported in this study is a causing factor or a consequence of the ER abnormalities in HD cannot be clarified here. However, defects in ER may conceivably affect enzyme activities. In this context, our findings let us to hypothesize a double mechanism by which HTT mutation may affects *de novo* sphingolipid synthesis. Data of gene expression obtained from both early manifest mice and *in vitro* model, support the hypothesis that reduced activity of SPT may represent the initial “toxic” event which is later followed by a transcriptional increase of sptlc1 and sptlc2 gene expression as compensatory mechanism.

From our perspective, we believe that our findings overall suggest that defects in the metabolism of sphingolipids and their *de novo* synthesis in HD cells may exacerbate cell dysfunction in the disease and may represent a plausible pathogenic factor rather than an epiphenomenon secondary to the worsening of the disease.

In conclusion, our study serves as first evidence of aberrant *de novo* biosynthetic pathway of sphingolipids in HD and, along with previous findings of altered sphingolipid metabolism (Di Pardo et al., 2017a), it provides more indications to consolidate the concept of perturbed lipid homeostasis in the disease and to identify potential and novel attractive target for the development of therapeutic strategies in HD.

## Author contributions

VM: conceived and designed the study; VM and ADP: jointly directed the study and co-wrote the manuscripts; ADP and FB: supervised all the *in vivo* experiments; SC and FM: managed animal colonies; GP: performed qPCR analysis in mouse tissues; EA: performed all the *in vitro* experiments; AD: managed HD fly models and performed qPCR experiments; AB and AA: performed Mass Spectrometry analysis; All the authors analyzed and discussed the data, revised and approved the manuscript.

### Conflict of interest statement

The authors declare that the research was conducted in the absence of any commercial or financial relationships that could be construed as a potential conflict of interest.
