# Supplementary material for: De novo Synthesis of Sphingolipids Is Defective in Experimental Models of Huntington's Disease
Source: Front Neurosci. 2017 Dec 19;11:698. doi: 10.3389/fnins.2017.00698 (PMC5742211; doi:10.3389/fnins.2017.00698)
Supplement: Supplementary file 1 [file DataSheet1.PDF]

Supplementary Information

***De novo* synthesis of sphingolipids is defective in Huntington's disease experimental models.**

Di Pardo A[1], Basit A[2], Armirotti A[2], Amico E[1], Castaldo S[1], Pepe G[1], Marracino F[1], Digilio AF[3], and Maglione V[1]*.

[1]IRCCS Neuromed, Pozzilli (IS), Italy

[2]Department of Drug Discovery and Development, Fondazione Istituto Italiano di Tecnologia, Genova, Italy

[3]Institute of Biosciences and Bioresources (IBBR), National Research Council (CNR), Naples, Italy

***Correspondence to:**

Vittorio Maglione

E-mail: vittorio.maglione@neuromed.it

Results

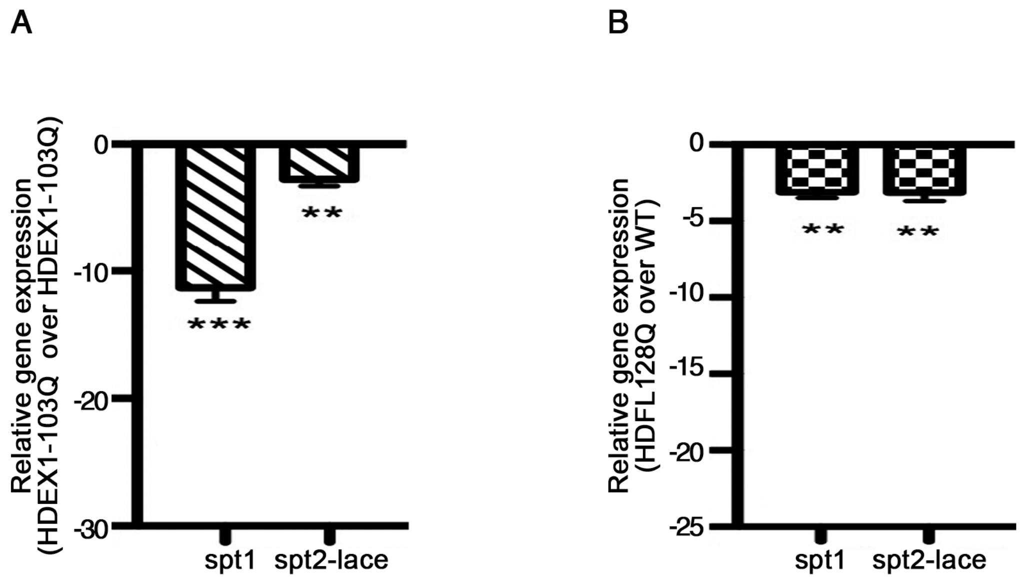

Supplementary Figure 1. Gene expression of both *spt1* and *2* is reduced in fly models of HD. Relative expression of sptcl1 (*spt1)* and sptcl2 (*spt2)* transcripts in 15 day old Drosophila expressing either hHtt exon1 with 103 polyglutamine (HDEX1-23Q) (**A**) or full length hHtt with 128 polyglutamine (HDFL-128Q) (**B**) and age matched wild type controls. Bars represented the average of three independent experiments each performed in triplicate. Values are mean ± SE. **p < 0.001, ***p< 0.0001, HD versus Controls, (Unpaired t-test).

Materials and Methods

Drosophila stocks and crosses. Flies were reared on standard cornmeal-agar and raised at 28°C. In all experiments, 15 days post- eclosion adult males and females were used. Fly stocks used in the present study were obtained from the Bloomington Stock center (w*; P [UAS-HTT.128Q.FL] f27b -Bloomington 33809-, w ;P [GAL4-elav.L] 2 -Bloomington 55635-), with the exception of P UAST-Httex1-Q103-eGFP and P [UAS-Httex1 Q23-eGFP] (a gift from Sheng Zhang).

Expression of polyglutamine-containing human huntingtin (hHtt) is driven by the bipartite expression system upstream activator sequence (UAS)-GAL4 in transgenic flies (Brand and Perrimon, 1993).

Flies bearing the hHtt constructs under the control of a yeast UAS were crossed to flies expressing the yeast GAL4 transcriptional activator driven by the neuron-specific promoter elav that is expressed in all neurons from embryogenesis onwards.

The genotypes of the flies used to express the hHtt constructs were as follows: P {UAS-Httex1 Q23-eGFP/P{GAL4-elav.L}2 as control strain for the flies P UAST-Httex1-Q103-

eGFP/P{GAL4-elav.L}2; P{UAS-HTT.128Q.FL}f27b/P{GAL4-elav.L}2 and the non - activated w*; P{UAS-HTT.128Q.FL}f27b as its control.

Fly tissues. Total RNA was isolated from 15 day old control and hHtt groups flies, using TRI Reagent (Sigma-Aldrich, Cat N. T9424) according to the manufacturer's instructions. Each group consisted of heads of 5 males and 5 females. The RNA integrity was checked by electrophoresis on a 1.0% agarose gel in TAE buffer and visualized under UV light. Absorbance ratio at 260/280 nm was also quantified using a spectrophotometer; and 500 µg RNA samples were put into a 20 µl reverse transcription (RT) reaction, using a SuperScript III First-Strand Synthesis System for RT-PCR (Invitrogen, Cat. N. 18080-051). cDNA was diluted ten-fold, 1 µl was used in 10 µl quantitative qPCR assays using SYBR Master Mix (Applied Biosystems, Cat. N. 4367659), and qPCR was done on an ABI 7900 HT Fast Real Time PCR system instrument. Each sample was normalized to *rp49* transcript levels and control samples. The following primers were used (5'—>3'): spt1Fw-CCAGCCCGCCGAGAAC; spt1Rv–AGCCACTCCACGAGCAATG; spt2Fw–GGCAGTGCTGGAGGCTATCT; spt2Rv TTGGTGCGGAGAAAGTCAATC; rp49RTFw–CAGTCGGATCGATATGCTAAGC; rp49RTRv –GGCATCAGATACTGTCCCTTGAA.
